# Detection of Significant Seismic Quiescence Patterns in the Mexican Subduction Zone Using Extended Schreider Algorithms

**DOI:** 10.3390/e27090960

**Published:** 2025-09-16

**Authors:** Carlos Carrizales-Velazquez, Jennifer Perez-Oregon, Israel Reyes-Ramírez, Lev Guzmán-Vargas, Fernando Angulo-Brown

**Affiliations:** 1 Laboratorio de Sistemas Complejos, Unidad Interdisciplinaria en Ingeniería y Tecnologías Avanzadas, Instituto Politécnico Nacional, Ciudad de México 07340, Mexico; ireyesr@ipn.mx; 2 Departamento de Ciencias Básicas, Universidad Autónoma Metropolitana, Azcapotzalco, Ciudad de México 02128, Mexico; jnnfr.po@gmail.com; 3 Departamento de Física, Escuela Superior de Física y Matemáticas, Instituto Politécnico Nacional, Ciudad de México 07738, Mexico; fangulob@ipn.mx

**Keywords:** seismic precursors, mexican subduction zone, schreider algorithm, seismic quiescence

## Abstract

This study investigates the implementation of Schreider’s quiescence algorithm and two variants that utilize spatiotemporal data to identify patterns of seismic quiescence. These patterns are of particular interest as they may serve as precursors to major seismic events, specifically large earthquakes (M>7), within the Mexican subduction zone associated with the Cocos Plate. We identify two characteristic stages: the α-stage, where a notable deviation is observed in the Schreider convolutions, and the β-stage, where the convolutions return to their background levels. In addition, we identify that the Schreider algorithm cannot discern quiescence patterns when earthquakes M>7 are too close in space and time. Consequently, we explore the behavior of the convolutions in three cases where the algorithm is restarted after the mainshocks, and we find apparent advantages for the spatiotemporal variants of the convolutions. The findings contribute to a more profound understanding of the stages preceding large subduction earthquakes and aid in the identification of precursor patterns in this region.

## 1. Introduction

Within the context of earth sciences, since several decades ago, the problem of earthquake prediction has attracted the attention of many researchers [1,2]. However, at the present time, no universally accepted method to predict the occurrence of large-magnitude earthquakes currently exists. Nevertheless, efforts to investigate this extraordinarily complex problem continue [3,4]. One of the methods most commonly used is searching for quiescence patterns before the occurrence of great earthquakes using different approaches such as those proposed by Ohtake [5,6,7] and Schreider [8], among many others [9,10,11,12,13,14,15,16]. In fact, Wyss and Habermann (1988) defined the quiescence pattern formally as follows [10]: “seismic quiescence is a decrease of mean seismicity rate as compared to the background rate in the same crustal volume, judged significant by some clearly defined standard. The rate decrease (α-stage proposed by Ohtake [5]) takes place within part, or all, of the source volume of the subsequent main shock, and it extends up to the time of the mainshock or may be separated from it by a relatively short period of increased seismicity rate (β-stage [5])”. For a pedagogical scheme, see the explanation presented online in Section S1 of the Supplementary Materials. However, the hypothesis of precursory seismic quiescence is not universally accepted, and there exist different approaches to measure, map, and evaluate possible episodes of seismic quiescence [17].

Nonetheless, the pursuit of quiescence patterns is regarded as a promising approach for approximating the forecast of great EQs. In the present article, the Schreider algorithm is employed in its original form and in a modified form. Specifically, this study unveils the existence of quiescence patterns derived from convolution series comprising spatiotemporal information. These patterns are poised to be associated with a series of eight earthquakes of magnitude M>7 that occurred along the Pacific coast of Mexico from 2012 to 2022. Furthermore, we employ a relatively novel method that was published in 2018. This method involves the exploration of a quantity referred to as seismic pseudo-velocity [18]. The present article is organized as follows: Section 2 is dedicated to reviewing the methods we use to study the possible seismic quiescence patterns along the Pacific Mexican Coast in the Latitude interval [10° N, 22° N] and in the Longitude interval [108° W, 87° W] during the period from 1 January 1990 until 1 January 2025. In Section 3, we describe the database used for this paper. In Section 4 and Section 5, we show the principal results of the application of the methods described in Section 2 to eight earthquakes (M>7); all of them presented evidence of quiescence patterns before the respective mainshocks. Finally, in Section 6, we present our concluding remarks.

## 2. Methods

### 2.1. Schreider Algorithm

The identification of zones of seismic quiescence can be facilitated by employing the Schreider algorithm [8]. The initial step entails the selection of a threshold magnitude, designated as $M_{th}$, and the delineation of a volumetric region for the study. In this study, cylindrical volumes of radii *r* are utilized, as previously defined in [14,18]. The focal mechanism of each mainshock is centered in a cylindrical region, with the objective of identifying significant anomalies in the inter-times ΔT(n)=t(n)−t(n−1) of consecutive earthquakes. In this context, t(n) denotes the occurrence time of the *n*-th seismic event. Consequently, ΔT(n) is defined as a measure of seismic frequency, with low (high) seismicity corresponding to large (small) values of ΔT, respectively. To ensure the desired level of smoothness, the Laplace function, denoted by f(n,s), is employed [8]). The definition of this function is as follows:

(1) $$f\left(n,s\right)=\frac{1}{s\sqrt{2\pi }}\exp\left[-\frac{n^{2}}{2s^{2}}\right],\;n=1,2,3,...,$$

where *s* is the *smoothness* parameter [8]. Subsequently, each $k_{th}$ earthquake can be associated with the convolution through the application of the following formula:

(2) $$T\left(k\right)=\sum_{n=0}^{l}\Delta T\left(k-n\right)f\left(n,s\right),$$

where the value of *l* is determined when the function f(n,s) is approximately zero. Consequently, the convolution T(k) is determined by three parameters, $M_{th}$, *r*, and *s*, which are selected on an arbitrary basis. In order to identify a period of seismic quiescence, it is necessary to ascertain the average value of T(k) calculated over an extended period of time, under the assumption that this average value is then stabilized. This average value $\bar{T}$ is as follows:

(3) $$\bar{T}=\frac{1}{N}\sum_{k=1}^{N}T\left(k\right),$$

where *N* is the total number of earthquakes with magnitude $M\geq M_{th}$ which fall inside the exploration of the cylindrical region. The standard deviation is calculated as follows:

(4) $$\sigma =\sqrt{\frac{1}{N-1}\sum_{k=1}^{N}{\left[T\left(k\right)-\bar{T}\right]}^{2}}.$$

The temporary convolution function T(k) follows a Gaussian distribution. Because of this, Schreider assumed that values of T(k) greater than $\bar{T}+3\sigma$ are considered abnormally large. Therefore, cylindrical regions that show this behavior are considered to contain abnormal seismic quiescence [14].

### 2.2. Analysis of Spatio-Temporal Series Using the Schreider Algorithm

The Schreider algorithm focuses exclusively on the temporal characteristics of seismic events, with the objective of identifying periods of seismic quiescence. We hereby propose the inclusion of the inter-distance, denoted here as ΔR, of consecutive earthquakes:

(5) $$\Delta R\left(n\right)=∥\vec{R}\left(n\right)-\vec{R}\left(n-1\right)∥,$$

where ∥·∥ denotes the euclidean distance. We hereby propose the implementation of the convolution utilized in the Schreider algorithm, albeit employing the product of inter-times and inter-distances, as outlined below:

(6) $$\mathrm{RT}\left(k\right)=\sum_{n=0}^{l}\left[\Delta R(k-n)\times \Delta T(k-n)\right]f\left(n,s\right).$$

Equation (6) includes the role of seismic frequency and spatial occurrence (distance between consecutive hypocenters) for searching seismic quiescence.

### 2.3. Analysis of Pseudo-Velocity Series Using the Schreider Algorithm

In the aforementioned work [18], the concept of *pseudo-velocity* was introduced with the objective of quantifying multifractal changes associated with major seismic events (M>7). We examine the behavior of this novel variable, which encompasses the temporal and spatial characteristics of seismic events. The pseudo-velocity is defined as the logarithm of the quotient of ΔR and ΔT [18]. Subsequently, the convolution for this new variable is defined as follows:

(7) $$V\left(k\right)=\sum_{n=0}^{l}log\left[\frac{\Delta R(k-n)}{\Delta T(k-n)}\right]f\left(n,s\right).$$

The results generated by the Schreider algorithm are typically on the order of ∼$\left[10^{4},10^{5}\right]$, as the inter-times are within these orders [14]. However, the values of pseudo-velocity series are in the lower range of magnitudes ∼$\left[10^{-5},10^{1}\right]$ [18]. As is evident, the values differ significantly, and a plot that incorporates both set of results is not visually appealing. Subsequently, the quantities ΔR and ΔT are normalized with their standard deviations σ(ΔR) and σ(ΔT), respectively. For clarity, the following notations will be employed: $\tilde{T}\left(k\right)$ and $\tilde{\mathrm{RT}}\left(k\right)$ for Equations (2) and (6), respectively, following the normalization procedure outlined herein. A Pipeline Diagram as a summary and complement to this section can be found online in the Section S2 of the Supplementary Materials.

## 3. Data: Southern Mexican Pacific Seismic Zone

The southern region of Mexico is characterized by a high degree of seismic complexity, which is attributable to the subduction zone where the Cocos, Pacific, and North American plates converge. This dynamic geological setting is a primary source of significant seismic activity in the region. The utilization of seismic data in this study has been facilitated by the National Seismological Service of Mexico (SSN) [19], which has maintained records since the early 20th century. It has been documented that the database is complete from 1990 onwards [18]. An endeavor has been undertaken to categorize the Mexican region based on its seismic dynamics [20].

## 4. Seismic Quiescence of Mexico Mainshocks, Unmixed Patterns

**Figure 1:**
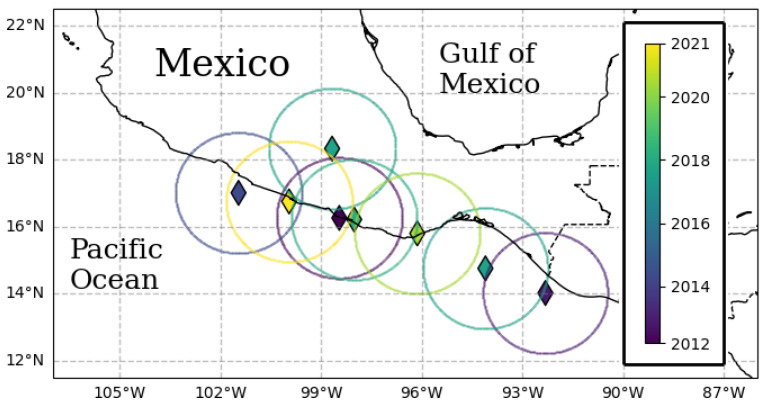
This map shows mainshocks (EQ>7) in Mexico during the period of 2012–2025. The epicenters are shown as diamonds, each with its circular study region.

For the purpose of this study, earthquakes of magnitude M>7 are considered mainshocks within the period from 1 January 2006 to 31 December 2024 (see Table 1). In accordance with the approach outlined in [18], this study focuses on cylinders with radii R=200 km and heights H=60 km. During the period under consideration, eight major seismic events were recorded. Figure 1 presents the epicenters of these seismic events, along with their respective radii and the date and time of occurrence. As observed, such radii exhibit overlapping regions that must be taken into account when interpreting seismic quiescence patterns accurately.

### 4.1. EQ 7.5, 20 March 2012

**Figure 2:**
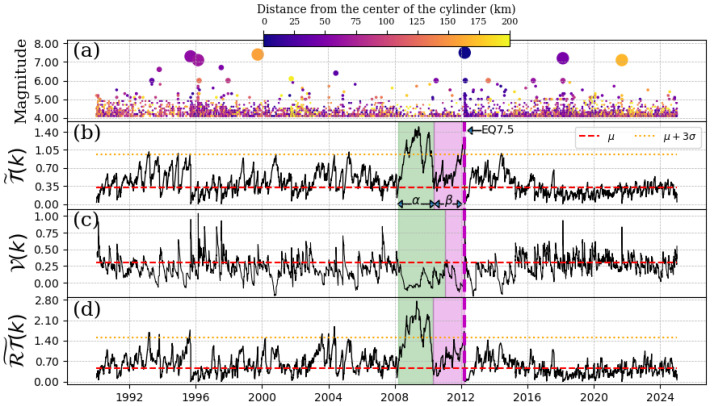
Schreider convolution corresponding to EQ7.5, 20 March 2012. (**a**) Time series of earthquake magnitudes in the cylindrical region with a threshold magnitude of $M_{th}=4.1$. The coloration of the circles is indicative of the distance between the center of the cylinder and the epicenter of each earthquake, while the size of the circles corresponds to the earthquake magnitude. (**b**) Inter-time series $\tilde{T}\left(k\right)$, (**c**) pseudo-velocity series V(k), and (**d**) the product of inter-distance with inter-time $\tilde{\mathrm{RT}}\left(k\right)$ series. Red dashed line shows the mean value of the convolution (for each case), orange dotted line is three times the standard deviation for each case, and shaded green and magenta regions correspond to the α and β stages, respectively. The cylinder used has a radius of 200 km and a depth within the range of [0, 60] km.

The seismic event in question occurred in the area between the municipalities of Ometepec-Guerrero and Pinotepa Nacional-Oaxaca. The depth of the cylinder ranges from 0 km to 60 km. Here, we consider a threshold magnitude, $M_{th}=4.1$. As illustrated in Figure 2, the time series of seismic events within the specified region of study has been examined, along with the outcomes of the Schreider algorithm’s implementation on inter-times, pseudo-velocities, and product inter-distance with inter-time series for the EQ7.5. Figure 2a presents the time series of the magnitudes. As we can see in Figure 2b,d, the values shown for $\tilde{T}\left(k\right)$ and $\tilde{\mathrm{RT}}\left(k\right)$ do not exceed threshold $\bar{T}+3\sigma$ until the beginning of 2008 (where the green shade begins). To determine the intervals at which the convolution consistently exceeds the $\bar{T}+3\sigma$ threshold, we used the method that detects the first return to the mean value [21] (see Section S3 of the Supplementary Materials for details). An increase was observed from the beginning of 2008, which lasted two years (considered the α-stage by [5]). The values of the convolution in Figure 2b,d returned to their initial behavior for approximately two years (considered the β-stage by [5]) until the EQ7.5 mainshock occurred. It is important to note that three main shocks occurred during the α-stage, but as they happened after EQ7.5, they possibly did not interfere with the quiescence shown in Figure 2. Regarding pseudo-velocity, as shown in Figure 2c, its values decreased and remained low throughout the entire α-stage period.

### 4.2. EQ 7.3, 7 November 2012

**Figure 3:**
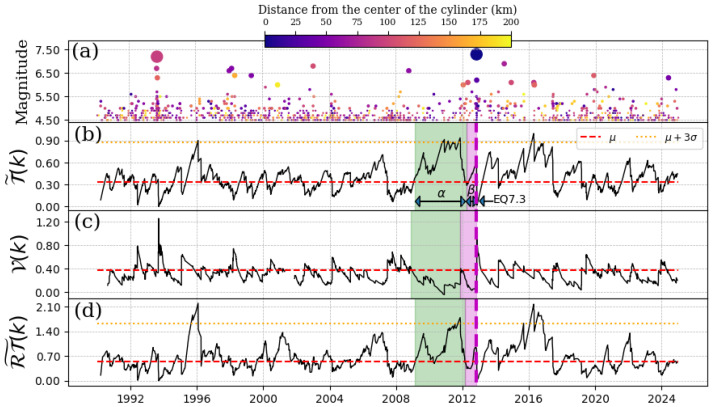
Schreider convolution for EQ7.3, 7 November 2012. (**a**) Time series of earthquake magnitudes in the cylindrical region with a threshold magnitude of $M_{th}=4.5$. The coloration of the circles is indicative of the distance between the center of the cylinder and the epicenter of each earthquake, while the size of the circles corresponds to the earthquake magnitude. (**b**) Inter-time series $\tilde{T}\left(k\right)$, (**c**) pseudo-velocity series V(k), and (**d**) the product of inter-distance with inter-time $\tilde{\mathrm{RT}}\left(k\right)$ series. Red dashed line shows the mean value of the convolution (for each case), orange dotted line is three times the standard deviation for each case, and shaded green and magenta regions correspond to the α and β stages, respectively. The cylinder used has a radius of 200 km and a depth within the range of [0, 60] km.

This earthquake happened close to the intersection between the Cocos and Caribbean tectonic plates [19]. Its epicenter (14.03 N, 92.32 W) was located in Ciudad Hidalgo-Chiapas at a depth of 17 km. For this case, the cylinder was set from 0 km to a depth of 60 km. The results are shown in Figure 3, using a threshold magnitude of $M_{th}=4.5$. As we can see, the deviations in the convolutions in Figure 3b–d show an anomaly close to the EQ7.3, but their values barely reach the limit 3σ. We identified brief α and β stages, which lasted approximately three years and half of a year, respectively. On the other hand, we observe a peak near the end of 1995 that could be associated with the EQ7.1, which occurred on 20 October 1995 at a latitude of 16.81° N and a longitude of 93.47° W. Additionally, an anomaly is observed after the mainshock of this subsection, which could be associated with the EQ8.2 on 7 September 2017.

### 4.3. EQ 7.2, 18 April 2014

**Figure 4:**
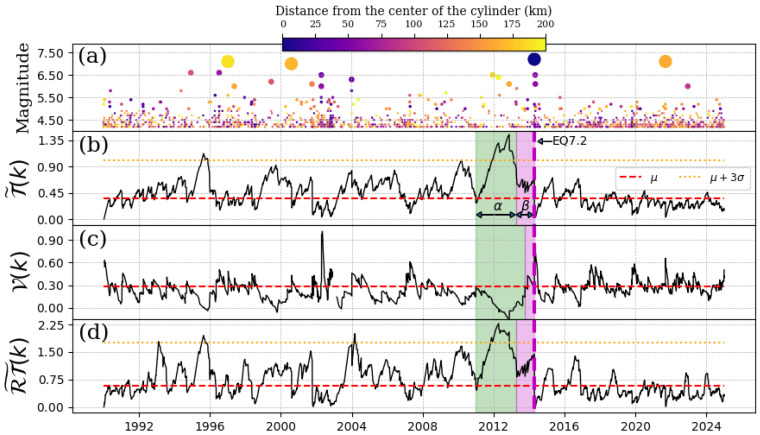
Schreider convolution for EQ7.2, 18 April 2014. (**a**) Time series of earthquake magnitudes in the cylindrical region with a threshold magnitude of $M_{th}=4.2$. The coloration of the circles is indicative of the distance between the center of the cylinder and the epicenter of each earthquake, while the size of the circles corresponds to the earthquake magnitude. (**b**) Inter-time series $\tilde{T}\left(k\right)$, (**c**) pseudo-velocity series V(k), and (**d**) the product of inter-distance with inter-time $\tilde{\mathrm{RT}}\left(k\right)$ series. Red dashed line shows the mean value of the convolution (for each case), orange dotted line is three times the standard deviation for each case, and shaded green and magenta regions correspond to the α and β stages, respectively. The cylinder used has a radius of 200 km and a depth within the range of [0, 60] km.

The mainshock of 18 April 2014 had its epicenter in Petatlán-Guerrero (17 N, 101.46 W). In this case, we also use the same depths for the cylinders as in the previous two reported earthquakes. Figure 4 shows an anomaly that begins at the end of 2011 and disappears at the beginning of 2014 (the α-stage, represented by the green shaded region). The β-stage lasted just a few months until the EQ7.2. This behavior was also reported when studying the long-range correlations of the electromagnetic signals of the ground near this epicenter in [22]. Additionally, we observe a significant increase in $\tilde{T}\left(k\right)$ in 1995, which may be primarily associated with the EQ7.1 (11 January 1997), whose epicenter was located at 189 km.

### 4.4. EQ8.2, 7 September 2017

**Figure 5:**
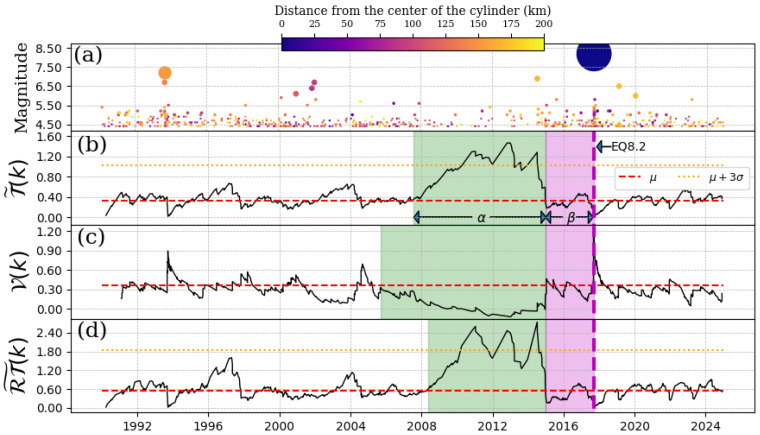
Schreider convolution for EQ8.2, 7 September 2017. (**a**) Time series of earthquake magnitudes in the cylindrical region with a threshold magnitude of $M_{th}=4.4$. The coloration of the circles is indicative of the distance between the center of the cylinder and the epicenter of each earthquake, while the size of the circles corresponds to the earthquake magnitude. (**b**) Inter-time series $\tilde{T}\left(k\right)$, (**c**) pseudo-velocity series V(k), and (**d**) the product of inter-distance with inter-time $\tilde{\mathrm{RT}}\left(k\right)$ series. Red dashed line shows the mean value of the convolution (for each case), orange dotted line is three times the standard deviation for each case, and shaded green and magenta regions correspond to the α and β stages, respectively. The cylinder used has a radius of 200 km and a depth within the range of [30, 90] km.

This is the greatest magnitude earthquake in this work, which had its epicenter in the Tehuantepec’s Gulf, Chiapas. In this case, the hypocenter was located at a depth of 46 km, and its cylinder ranged from 30 km to 90 km. This event is one of the cases reported in [18] (threshold magnitude $M_{th}=4.4$), but here, we provide additional information by applying the Schreider algorithm to the pseudo-velocities and the product $\tilde{\mathrm{RT}}\left(k\right)$ series. Figure 5 shows the possible quiescence presented before the EQ8.2. In Figure 5a, we reproduce the behavior reported previously in [18]. Figure 5c shows a more statistically significant pattern where a decreasing behavior is observed in the pseudo-velocities, which also lasted from 2008 until beginning of 2015. This agrees with the so called α-stage showed in Figure 5b (shaded green region). The behavior shown in Figure 5d coincides with Figure 5b.

### 4.5. EQ7.1, 19 September 2017

**Figure 6:**
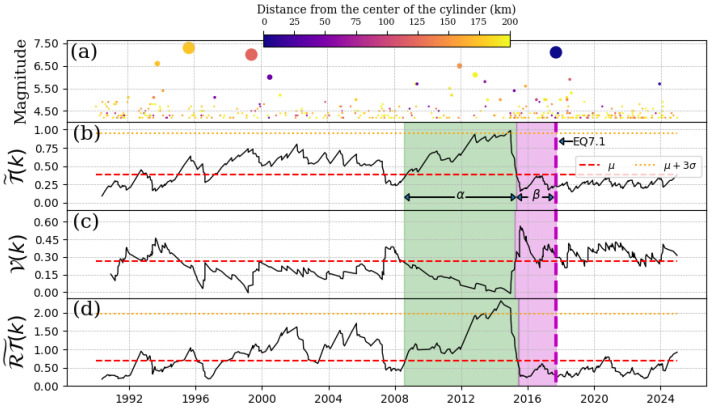
Schreider convolution for EQ7.1, 19 September 2017. (**a**) Time series of earthquake magnitudes in the cylindrical region with a threshold magnitude of $M_{th}=4.2$. The coloration of the circles is indicative of the distance between the center of the cylinder and the epicenter of each earthquake, while the size of the circles corresponds to the earthquake magnitude. (**b**) Inter-time series $\tilde{T}\left(k\right)$, (**c**) pseudo-velocity series V(k), and (**d**) the product of inter-distance with inter-time $\tilde{\mathrm{RT}}\left(k\right)$ series. Red dashed line shows the mean value of the convolution (for each case), orange dotted line is three times the standard deviation for each case, and shaded green and magenta regions correspond to the α and β stages, respectively. The cylinder used has a radius of 200 km and a depth within the range of [0, 90] km.

This earthquake happened on the border of the states of Puebla and Morelos, and its consequences on Mexico City were devastating. It is important to mention that this region was not very seismically active before this EQ; consequently, the seismic rate of this region increased due to the aftershocks, and this zone reached a new seismicity level. The depth of the hypocenter was 51.2 km; that is, the epicenter was far from the so-called Pacific Trench, inland from the continental plate. For this case, we chose a cylinder with depth from 0 km to 90 km. We observe convolutions (see Figure 6) that barely reach $\bar{T}+3\sigma$ at the beginning of 2012, and it decays at middle of 2015, as observed in Figure 6b, which could be considered as the α-stage. Next, the β-stage lasted two and a half years until the mainshock. Some of this happens when $\tilde{\mathrm{RT}}\left(k\right)$ is combined with other things (see Figure 6d). Actually, for this EQ, the more important quantity was the convolution of pseudo-velocities (see Figure 6c) It is noteworthy that this region typically exhibits low seismic activity, which complicates the discernment of quiescence periods.

## 5. Seismic Quiescence of Mexico Mainshocks, Possibly Mixed Patterns

As Figure 1 shows, the last three EQs on the list occurred on dates that were approximately one to two years apart. Additionally, their epicenters were relatively close to each other. This could cause their quiescence patterns to overlap, considering the typical duration of individual quiescence patterns. For this reason, we discuss these three cases separately from the previous ones.

### 5.1. EQ7.2, 16 February 2018

**Figure 7:**
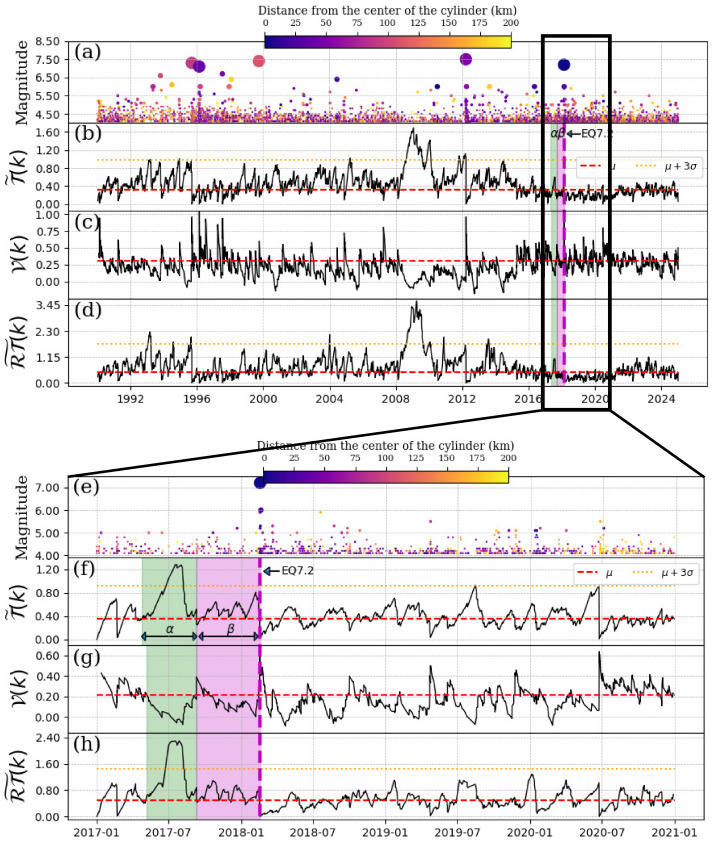
Schreider convolution for EQ7.2, 2018-02-16. (**a**) Time series of earthquake magnitudes in the cylindrical region with a threshold magnitude of $M_{th}=4.1$. The coloration of the circles is indicative of the distance between the center of the cylinder and the epicenter of each earthquake, while the size of the circles corresponds to the earthquake magnitude. (**b**) Inter-time series $\tilde{T}\left(k\right)$, (**c**) pseudo-velocity series V(k), and (**d**) the product of inter-distance with inter-time $\tilde{\mathrm{RT}}\left(k\right)$ series. The cylinder used has a radius of 200 km and a depth within the range of [0, 60] km. Panels (**e**–**h**) zoom in on a segment of the time series shown in panels (**a**–**d**) (above). The red dashed line shows the mean of the convolution of each series, the orange dotted line is three times the standard deviation of the new segment, the magenta dashed vertical line indicates the date of the mainshock, the shaded green and magenta regions corresponds to the α and β stages, respectively.

This earthquake happened near the town Pinotepa Nacional, Oaxaca at a depth of 16 km. We chose a cylinder from 0 km to 60 km in depth. It is important to mention that this event was located at 47.6 km from the EQ7.5 of 20 March 2012 (see Figure 1). In consequence, the quiescence of the EQ7.2 could be veiled with the quiescence of the EQ7.5. Figure 7b–d show the resulting series of convolutions using the Schreider algorithm. As we can see, the most notable deviation pattern is observed from 2008 until 2012; however, this period corresponds to the quiescence of the event EQ7.5, as we just commented. Unfortunately, we do not identify important changes in the convolution of $\tilde{T}\left(k\right)$, but in the convolution of the pseudo-velocity V(k) and the $\tilde{\mathrm{RT}}\left(k\right)$ series, we observe small changes (α and β regions shown in Figure 7c,d), which could be associated with seismic quiescence of the event EQ7.2 on 16 February 2018. Including the spatial aspect in the Schreider convolutions could be an important advantage to recognize possible seismic quiescence just after a mainshock, which could hide changes in the classical convolution for shortly after the mainshocks of inter-times $\tilde{T}\left(k\right)$. As we assumed, the previous mainshock EQ7.5 produces a kind of veil for the quiescence pattern. Then, we decided to re-run the analysis, but beginning after the aftershocks produced by the previous EQ7.5 (a few of years after the mainshock). The results of these new calculations are shown in Figure 7f–h. Here, we observe how the 3σ value of the convolution decreases. Consequently, a new seismic quiescence pattern rises, which corresponds to the mainshock in this section (EQ7.2, 16 February 2018) becoming clear. The α-stage begins on 20 May 2017 and ends on 10 September 2017 (green shaded region of Figure 7f–h). The β-stage lasted almost 5 months.

### 5.2. EQ7.4, 23 June 2020

**Figure 8:**
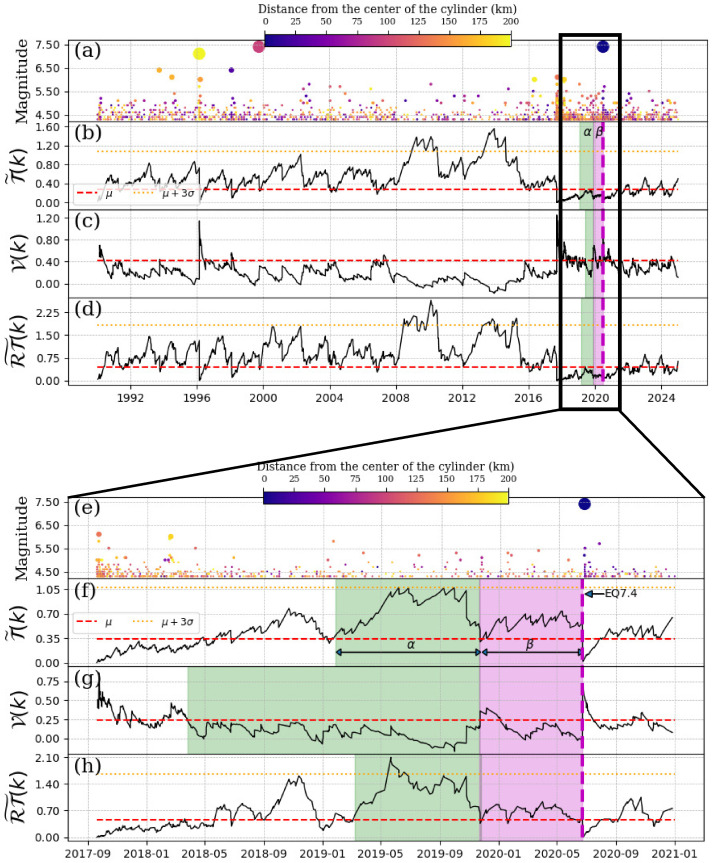
Schreider convolution for EQ7.4, 2020-06-23. (**a**) Time series of earthquake magnitudes in the cylindrical region with a threshold magnitude of $M_{th}=4.3$. The coloration of the circles is indicative of the distance between the center of the cylinder and the epicenter of each earthquake, while the size of the circles corresponds to the earthquake magnitude. (**b**) Inter-time series $\tilde{T}\left(k\right)$, (**c**) pseudo-velocity series V(k), and (**d**) the product of inter-distance with inter-time $\tilde{\mathrm{RT}}\left(k\right)$ series. The cylinder used has a radius of 200 km and a depth within the range of [0, 60] km. Panels (**e**–**h**) zoom in on a segment of the time series shown in panels (**a**–**d**). The red dashed line shows the mean of the convolution of each series, the orange dotted line is three times the standard deviation of the new segment, the magenta dashed vertical line indicates the date of the mainshock, the shaded green and magenta regions corresponds to the α and β stages, respectively.

Figure 8 shows similar behavior to that observed in the previous section (see Figure 7). We will then discuss Figure 8f–h, which begin on 1 September 2017. The Schreider convolution of inter-times (Figure 8f) reaches the 3σ values during the 9-month α-stage period (green shaded region in Figure 8f). The β-stage lasted 7 months (magenta shaded region in Figure 8f. In relation to the convolution of pseudo-velocity (Figure 8g), we observe that during the α-stage, this parameter decreases, as expected. In fact, in this α-stage, the decrement below the mean value is more remarkable than that observed in the β-stage. In Figure 8h, during the α-stage, the values of the convolution of ΔT×ΔR exceeded the 3σ-value.

### 5.3. EQ7.1, 7 September 2021

**Figure 9:**
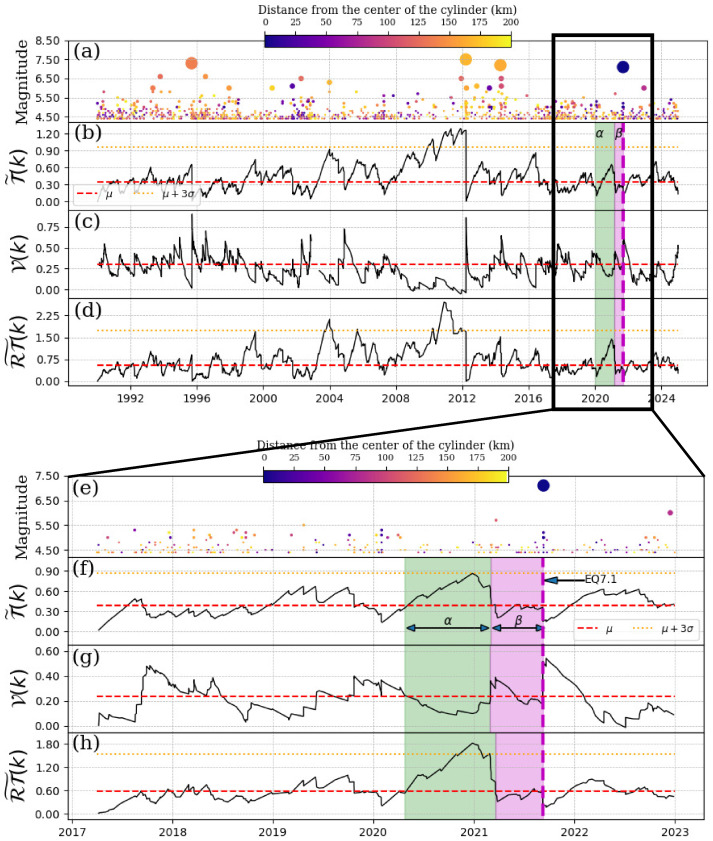
Schreider convolution for EQ7.1, 7 September 2021. (**a**) Time series of earthquake magnitudes in the cylindrical region with a threshold magnitude of $M_{th}=4.4$. The coloration of the circles is indicative of the distance between the center of the cylinder and the epicenter of each earthquake, while the size of the circles corresponds to the earthquake magnitude. (**b**) Inter-time series $\tilde{T}\left(k\right)$, (**c**) pseudo-velocity series $\tilde{V}\left(k\right)$, and (**d**) the product of inter-distance with inter-time $\tilde{\mathrm{RT}}\left(k\right)$ series. The cylinder used has a radius of 200 km and a depth within the range of [0, 60] km. Panels (**e**–**h**) zoom in on a segment of the time series shown in panels (**a**–**d**). The red dashed line shows the mean of the convolution of each series, the orange dotted line is three times the standard deviation of the new segment, the magenta dashed vertical line indicates the date of the mainshock, the shaded green and magenta regions corresponds to the α and β stages, respectively.

Another case of possible mixed patterns is the event EQ7.1 that occurred on 7 September 2021 (see Figure 9). As we can see, both the α and β stages are thin. We believe that this pattern was affected by the previous events that occurred: EQ7.5,2012; EQ7.2,2014; EQ7.1,2017; and EQ7.2,2018. All of these events had epicenters located almost 200 km from the epicenter of the EQ7.1 on 7 September 2021. In particular, the event EQ7.2,2018 is the closest event in time to the EQ7.1,2021. As Figure 9f–h show, before 2018, we can identify notable anomalies in the three convolutions, which correspond to all previous mainshocks. Due to these facts, we again apply the procedure mentioned in the previous subsections, which consists of beginning the analysis just after the last mainshock (EQ7.2,2018). In Figure 9f–h, we can see the results of this procedure. Now, the α and β stages for the EQ7.1, 7 September 2021 mainshock, are more clearly visible for the three convolutions.

The eight earthquakes presented in this article were thoroughly analyzed after they occurred. However, the procedure used (based on the Schreider method [8] with modifications proposed by us) can also be applied a priori in seismically active zones. We published a first step with a predictive approach in 2015 [14]. In that article [14], we investigated seismic quiescence over a period of nearly 27 years, observing 19 regions of quiescence, only three of which were significant precursors. In this same paper, however, we proposed a clear criterion to identify false alarms involving quiescence period durations and the level of convolutions considered statistically significant (see [14]).

### 5.4. Statistical Significance

We also evaluate the significance of our findings using the following method: We generate 1000 surrogates by randomly shuffling the inter-time, pseudo-velocity, and distance-time sequences and calculate the 95% confidence band. A state of persistent quiescence is identified if it exceeds the μ+3σ threshold and persists for a minimum of three successive intertimes. We confirm that, for randomized data from inter-times from all cases analyzed, the average values of $\tilde{T}\left(k\right)$ show relatively constant behavior, indicating uncorrelated temporal variation. For reference, see Section S4 of the Supplementary Materials; We observe that, in all cases, the actual data are clearly outside the confidence band, and $\tilde{T}\left(k\right)$ fully intersect with the confidence band in regions outside the alpha and beta periods. This indicates that these time intervals are indistinguishable from the surrogate distribution. Other details of the statistical analysis performed can be seen online in the Supplementary Materials.

## 6. Concluding Remarks

Without contradicting the affirmations of seismology that earthquakes cannot be predicted, our results provide evidence of quiescence patterns which were associated with eight earthquakes of magnitude M>7 on the Pacific Coast of Mexico from 2012 until 2022. The new implementation of *pseudo-velocity*V and *Time-Space* function RT as series for Schreider convolution provides new features that complement the original Schreider algorithm [8]. Our results regarding temporal convolution are in general agreement with those reported in studies of the Central Iran region [23]. For the case of pseudo-velocity convolution series V, we note that most of the time their values are below the mean $\bar{V}$ until a quiescence pattern appears. Here, the convolution of pseudo-velocity decreases relative to its mean value $\bar{V}$, which agrees with the Schreider hypothesis [8] identifying a decreasing seismic rate for a temporal period (the α-stage defined by Ohtake [5]) as a seismic precursor. Then, when the mainshock occurs, a brief increase of V happens, and its values return to its initial behavior. This feature was consistently observed in all the cases reported in this work, and we also noted that its dynamics were recovered almost instantaneously, showing the fading of the observed anomaly. If we compare this result with the convolutions T and RT, this could be considered an advantage in discriminating when a seismic quiescence pattern ceases or if an immediate quiescence pattern is observed. On the other hand, the convolution related to time and space RT shows a generally similar behavior to that observed for the original convolution T. Nevertheless, this new convolution RT revealed new features such as a (slightly) thinner form of the quiescence pattern, and in some cases, the values of RT exceeded the level μ+3σ of this convolution in such a way that it was clearer than that observed for convolution T.

In particular, the benefit of including the Schreider modifications (spatiotemporal variants) is that the threshold value of μ+3σ is more clearly exceeded by the $\tilde{RT}\left(k\right)$ convolutions for three of the EQs studied here: the EQ7.1, 19 September 2017; the EQ7.4, 23 June 2020; and the EQ7.1, 19 September 2017. On the other hand, by using the criteria of first-passage time [21] in the convolutions of pseudo-velocity, the durations of the α and β stages were larger and shorter, respectively, for the EQ8.2, 7 September 2017 and the EQ7.4, 23 June 2020. A tabular summary is presented online in the Section S3 of the Supplementary Materials. We assume these advantages are also due to the decrease in the seismic activity, where inter-distance ΔR plays an important role in the mechanism that possibly causes the seismic quiescence. In addition, by following the procedure of [18], we calculate the three Schreider algorithm versions presented for magnitude-threshold $M_{th}$ in the range [4.0, 4.5] for a radius of the cylindrical region in the range [150 km, 300 km] and for smooth parameter *s* in the range [3,27]. The results can be seen online in Section S5 of the Supplementary Materials; In all cases, the seismic quiescence patterns were observed satisfactorily. It is important to point out that the efficacy of the Schreider algorithm depends strongly on the selection of the threshold magnitude, $M_{th}$, and the size of the cylinder, which could vary from region to region. Because of this, the ranges of the parameters used in this study may not necessarily apply to other seismic zones in the world. On the other hand, the earthquake magnitude plays a critical role in different studies in the determination of seismic quiescence patterns preceding large events [17,24,25,26,27,28,29]. For the case of the Schreider algorithm, the earthquake magnitude role is part of the filtration procedure for the detection of seismic quiescence. Given the absence of specification in the seismic catalog utilized in this study regarding the magnitude type reported, a discussion is presented regarding the potential impact of the utilization of disparate magnitude scales on the outcomes of the Schreider algorithm. This discussion is accessible in the Section S7 of the Supplementary Materials; We must emphasize that using the same seismic scale would make the results of different seismic studies more accurate (see [30] for a detailed discussion of the use of different scales). However, this depends on the seismic catalogs, which do not always specify the type of magnitude scale. Nonetheless, we plan to explore the effects of homogenizing magnitude scales in future work. An important point observed in this work is the finding of possibly mixed patterns. The idea of using the Schreider algorithm just after a mainshock has not been sufficiently explored. Here, we proposed to restart the calculation of the Schreider algorithm; however, this implies that the data has an insufficient temporal duration for detecting the new background seismicity level of the studied region. We remark that earthquakes cannot be predicted, in the sense that neither the exact time of occurrence nor the precise location can be predicted. However, this work provides evidence that seismic precursors could be considered as a complement to the evaluation of the short-term seismic hazard. Future work could enhance or strengthen Schreider’s algorithm by integrating modern spatiotemporal data mining methods that better capture the complex spatial and temporal dependencies of seismic data. For example, better use of cell-based spatial segmentation combined with time-sequence analysis could allow for more accurate detection of quiescence patterns in distinct regions with significant seismic activity. Seismic precursors have been found in seismic data, electric and magnetic fields, gas/aerosol emissions, outgoing long-wave radiation, air temperature anomalies, geochemical changes, surface deformations, and unusual animal behavior [22,31,32,33,34,35,36,37,38,39,40,41]. Machine learning techniques have been used to identify seismic features for seismic prediction and/or earthquake forecasting [42,43]. The seismic hazard forecast has been explored using spatial and temporal features of earthquake catalogs, with satisfactory results [44]. The main limitation is the data training, which generally has only a few major events (M > 7) [45], and the reported seismic periodicity for large earthquakes is too long [46]. Recent efforts for seismic quiescence have used techniques such as the ZMAP method [47], the Poisson probability map PMAP [48], anomalies using the ETAS model [49], and the RTL method [50,51]. There are also diverse techniques for identifying seismic quiescence [52]. Our findings contribute to these methods for studying seismic hazards.

## Figures and Tables

**Table 1:** List of mainshocks in Mexico (from 2006 until 2025).

Date	Magnitude	Latitude	Longitude	Depth (km)
20 March 2012	7.5	16.26	−98.46	18.0
7 November 2012	7.3	14.03	−92.32	17.1
18 April 2014	7.2	17.01	−101.46	18.0
7 September 2017	8.2	14.76	−94.10	45.9
19 September 2017	7.1	18.33	−98.67	51.2
16 February 2018	7.2	16.22	−98.01	16.0
23 June 2020	7.4	15.80	−96.13	22.8
17 September 2021	7.1	16.75	−99.95	15.0

## Data Availability

The data used in this study are available from the official website of the Servicio Sismológico Nacional de la Universidad Nacional Autónoma de México at www.ssn.unam.mx (last accessed date: 6 March 2025). The Python (version 3.12.3) code for the calculation of the original and modified version of the Schreider algorithm presented in this work can be found at: https://github.com/carrizales90/Schreider-Algorithm (last accessed date: 10 September 2025).

## Supplementary Materials

The following supporting information can be downloaded at: https://doi.org/10.6084/m9.figshare.29874155.v5 last accessed on 10 September 2025. References [53,54,55] are citied in the Supplementary Materials.

## Abbreviations

EQ(s)	Earthquake(s)
